# The effect of an endovascular Heaney maneuver to achieve total hepatic isolation on survival, hemodynamic stability, retrohepatic bleeding, and collateral flow in a porcine model

**DOI:** 10.1007/s00068-024-02482-2

**Published:** 2024-03-08

**Authors:** Maria B. Wikström, Anna Stene-Hurtsén, Jens Åström, Tal M. Hörer, Kristofer F. Nilsson

**Affiliations:** 1 Department of Emergency, Arvika Hospital, Region Värmland, Arvika, Sweden; 2 School of Medical Sciences, Örebro University, Örebro, Sweden; 3 Department of Cardiothoracic and Vascular Surgery, Department of Surgery, Örebro University Hospital and Faculty of Medicine and Health, Örebro University, Örebro, Region Örebro Län, Sweden; 4 Department of Anesthesiology and Intensive Care, Falun Hospital, Falun, Region Dalarna, Sweden

**Keywords:** Trauma, Retrohepatic inferior vena cava, REBOA, REBOVC

## Abstract

**Purpose:**

Combining resuscitative endovascular balloon occlusion of the aorta (REBOA) and the inferior vena cava (REBOVC) with open surgery is a new hybrid approach for treating retrohepatic vena caval injuries. We compared endovascular total hepatic isolation with supraceliac REBOA ± suprahepatic REBOVC and no occlusion in experimental retrohepatic vena cava bleeding regarding survival, bleeding volume, hemodynamic stability, and arterial collateral blood flow.

**Methods:**

Twenty-five anesthetized pigs (*n* = 6–7/group) were randomized to REBOA; REBOA + REBOVC; REBOA + infra and suprahepatic REBOVC + portal vein occlusion (endovascular Heaney maneuver, four-balloon-occlusion, 4BO) or no occlusion. After balloon inflation, free bleeding was initiated from an open sheath in the retrohepatic vena cava. Bleeding volume, right internal thoracic artery (RITA) blood flow, hemodynamics, and arterial blood variables were measured until death or up to 90 min.

**Results:**

The REBOA group had a longer median survival time (63 min) compared with the 4BO (24 min, *P* = 0.02) and no occlusion (30 min, *P* = 0.02) groups, not versus the REBOA + REBOVC group (49 min, *P* > 0.05). The first 15 min accumulated bleeding was comparable in all groups (*P* > 0.05); Thereafter, bleeding volume was higher in the REBOA group versus the 4BO group (*P* < 0.05), not versus the other groups. RITA blood flow and MAP were higher in the REBOA group versus the other groups after 10 min of bleeding (*P* < 0.05).

**Conclusions:**

Endovascular Heaney maneuver was not beneficial for survival or hemodynamic stability in this porcine model, whereas supraceliac REBOA was. Anatomical differences in thoracoabdominal collaterals between pigs and humans must be considered when interpreting these results.

**Supplementary Information:**

The online version contains supplementary material available at 10.1007/s00068-024-02482-2.

## Background

Despite improvements in pre- and intra-hospital care over the past decades, the mortality of patients with retrohepatic inferior vena cava (IVC) injuries remains high and largely unchanged [1–4]. In a retrospective report from Los Angeles in the 1990s concerning 12 patients with retrohepatic IVC injuries, all 12 patients died [1]. Although rare, retrohepatic IVC injuries continue to challenge trauma teams worldwide [1–6]. The IVC is a fragile vessel located behind the liver and difficult to access surgically. Surgical mobilization of the retrohepatic space may transform a contained retrohepatic hematoma into uncontrollable hemorrhagic shock [3–5, 7]. Retrograde bleeding from the right atrium, the hepatic veins, and other venous collaterals and blood inflow from the lower body may complicate management [4]. The “Pringle maneuver” involves temporary external occlusion of the portal vein and the hepatic artery and is sometimes combined with infra and suprahepatic vena cava and aortic cross clamping creating total hepatic vascular isolation, i.e., “The Heaney maneuver” [4]. These maneuvers are still commonly used in open surgical management of retrohepatic IVC injuries [4, 5]. However, despite these maneuvers, there may be collateral blood flow to the “isolated” retrohepatic injury site since the suprahepatic clamp is applied above the hepatic veins and may allow the inferior phrenic veins to empty into the retrohepatic space [4]. Endovascular balloon occlusion methods could help to achieve proximal and distal vascular control before entering the retroperitoneal hematoma, thus providing a potential advantage for the facilitation of open surgical definitive repair [3, 4, 7–13]. Combined endovascular and open surgery management is part of EndoVascular resuscitation and Trauma Management (EVTM); case reports of successful use of resuscitative endovascular balloon occlusion of the aorta (REBOA) + resuscitative endovascular balloon occlusion of the vena cava (REBOVC) combined with open surgery for IVC injuries in humans have been published [4, 7, 8, 13, 14].

We performed a completely endovascular “Heaney maneuver,” a method that has not been previously described elsewhere. This involved endovascular occlusion of the aorta (by supraceliac REBOA), the infra and suprahepatic vena cava (by two REBOVCs), the hepatic artery (by supraceliac REBOA), and the portal vein (by a portal vein occlusion balloon), i.e., “the four balloon occlusion” model (4BO). We opted not to apply the Pringle maneuver by laparotomy but rather examine if a comparable vascular occlusion could be achieved by alternative means, i.e., completely endovascular, since this was unknown to us. The main aim was to study how this combination affected retrohepatic bleeding and survival compared with only REBOA, REBOA + suprahepatic REBOVC, and no occlusion (control group)**.** Secondary aims were the study of collateral blood flow in the right internal thoracic artery (RITA), central hemodynamics, and blood gas markers of metabolism. We hypothesized that the 4BO would provide hemodynamic stability and bleeding control, thus prolonging survival time.

## Methods

### Animals

This randomized, prospective porcine study was performed in a University Hospital animal research laboratory. In total, 37 pigs were used. The animals were a cross between England Yorkshire, Swedish country breed, and Hampshire, aged 3 months, gender ratio 1:1, and mean weight 29.1 kg, range 23–33 kg. The Regional Ethical Committee (registration numbers 1660 and 041612–2022) approved the study before initiation. The study adhered to the ARRIVE guidelines, and the experiments were controlled by a veterinarian and supervised by an experienced scientist trained in animal laboratory research [15].

### Anesthesia

The animals received an intramuscular injection of 240 mg azaperone (40 mg/ml, Virbac, Kolding, Denmark) before transfer to the laboratory. On arrival at the laboratory, a mixture of zolazepam (6 mg/kg, Virbac), tiletamine (6 mg/kg, Virbac), and azaperone (4 mg/kg) was injected i.m to induce anesthesia. Atropine (1.5 mg i.m, Mylan, Stockholm, Sweden) was given prior to endotracheal intubation. General anesthesia was maintained using continuous infusions of propofol (10 mg/kg/h, Fresenius Kabi) and remifentanil (0,5 µg/kg/min, Meda AB, Solna, Sweden). The animals were mechanically ventilated in volume-controlled mode (tidal volume of 10 ml/kg), and the respiratory frequency was adjusted to normoventilation. A solution of 5% glucose (1 ml/kg/h, Fresenius Kabi) was infused continuously throughout the experiment. An infusion of Ringer’s acetate solution (10 ml/kg/h, Fresenius Kabi) was started during the initial preparations and stopped when the experimental retrohepatic bleeding began. Thermal blankets were used to keep body temperature at 37.5–39.5 °C. The animals were euthanized after completion of the experiments with an i.v dose of potassium chloride 40 mmol given rapidly in general anesthesia, and euthanasia was confirmed by asystole on electrocardiogram.

### Surgical preparation

A 10 Fr sheath was introduced into the right external jugular vein, and a 7.5 Fr Swan-Ganz arterial pulmonary catheter (Edward Lifesciences, Swan-Ganz CCOmbo, Irvine, CA, USA) was inserted to measure central venous pressure (CVP), semi-continuous cardiac output (CO), and body temperature. A 5 Fr sheath was placed in the right common carotid artery for sampling of arterial blood and measuring systemic blood pressure (SBP), mean arterial pressure (MAP), and heart rate (HR). A 7 Fr sheath was placed in the left external jugular vein for the administration of drugs and fluids. The right internal thoracic artery (RITA) was exposed by making a parasternal incision at the level of the 2nd intercostal space, and a 3 mm probe (Transonic Systems Inc, NY, USA) was placed for blood flow measurement. The splenic vein was exposed by an incision in the left flank, and an 11 Fr sheath was placed to prepare for the endovascular balloon occlusion of the portal vein. An 11 Fr sheath was placed in the right femoral artery for the REBOA catheter, and an 11 Fr sheath was placed in the right femoral vein for the suprahepatic REBOVC catheter. An 11 Fr sheath was placed in the left femoral vein for the infrahepatic REBOVC catheter. Through an incision in the right flank, an 11 Fr sheath with a side port tubing ending with a three-way stopcock was placed by the Seldinger technique in the retrohepatic vena cava with the tip of the sheath slightly below the hepatic veins. The three-way stopcock was positioned in a plastic bowl at heart level on a scale. The free bleeding was started by opening of the three-way stopcock at the beginning of the bleeding phase of the experiment. Then, a 10 mm probe (Transonic Systems Inc, NY, USA) was placed on the distal aorta, between the renal arteries and the bifurcation, to measure the distal aortic blood flow. A urinary catheter was placed in the urinary bladder using a small suprapubic incision.

### Study protocol

**Fig. 1:**
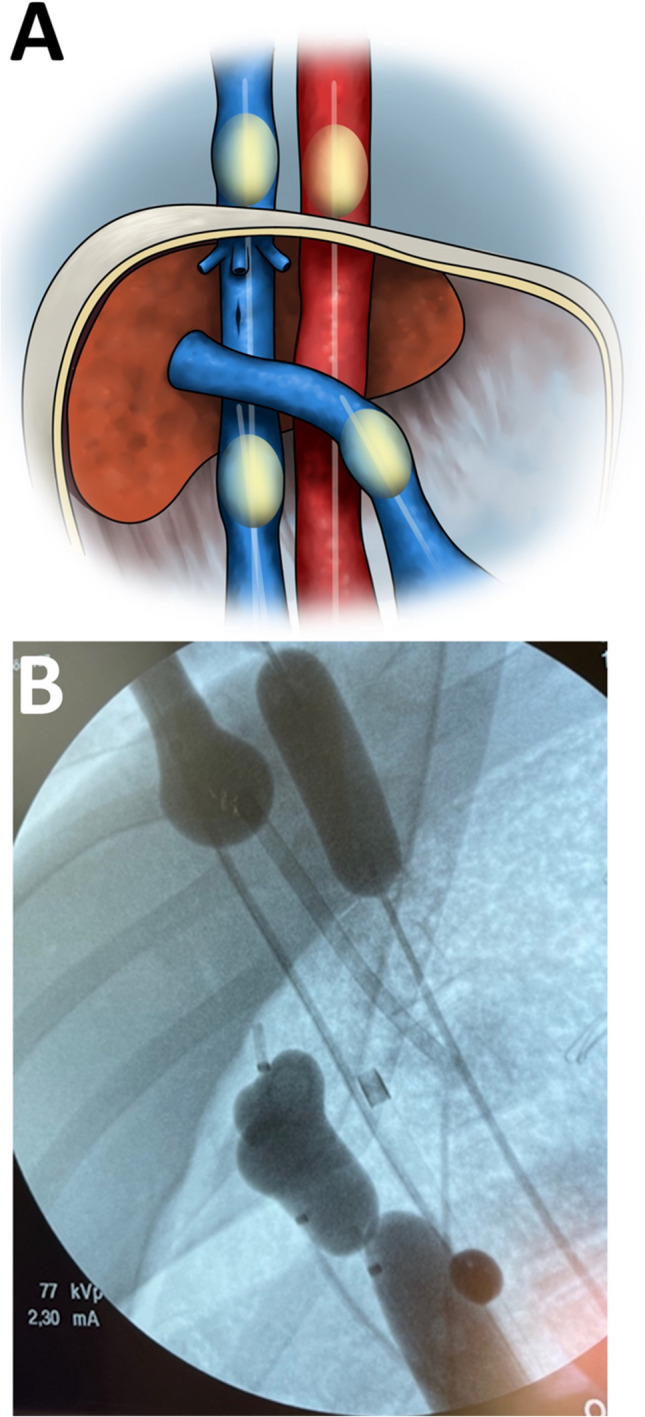
An illustration (**A**) and an x-ray (**B**) of the endovascular Heaney maneuver (four balloon occlusion) theoretically creating total hepatic isolation

When basic surgical preparations were finished, the animals received 5000 E Heparin i.v and were allowed to rest for 1 h. The animals were randomized by blindly drawing lots from a ballot to one of four groups: (1) supraceliac REBOA, (2) supraceliac REBOA + suprahepatic REBOVC, (3) supraceliac REBOA + infra and suprahepatic REBOVCs + portal vein balloon occlusion (four-balloon-occlusion, 4BO, Fig. 1), and (4) no occlusion (control group). The supraceliac REBOA was always inflated first, followed by occlusion of the portal vein, the infrahepatic vena cava, and lastly the suprahepatic vena cava. Correct balloon position was verified by fluoroscopy, and, in some animals, portal venography confirmed that the portal vein balloon did not occlude the splenic and superior mesenteric veins. We chose to use the Equalizer™ (Boston Scientific, Ireland) for venous occlusions based on previous experience with the Equalizer™ balloon for vena cava occlusion. For aortic occlusion, we used either the ER-REBOA™ (Prytime Medical, USA) or the Tokai™ balloon (Tokai Medical Products, Japan) [16, 17].

It took 5 min to inflate all balloons (− 5 min from start of bleeding = time 0). Balloon and sheath (in the retrohepatic vena cava) positioning and complete balloon inflation were intermittently checked during the bleeding phase using fluoroscopy. Ceased distal aortic blood flow was used as a marker for complete REBOA inflation. Immediately after balloon inflation, the infusion of Ringer’s acetate was stopped, 10,000 E Heparin was given, and free bleeding was started by opening of the three-way stop cock of the 11 Fr sheath in the retrohepatic vena cava. The pigs were placed in the left lateral position, and free bleeding was allowed from the side port of the sheath into a plastic bowl placed on a scale. The weight of blood in the plastic bowl and the blood flow in the RITA and the distal aorta were recorded at 1-min intervals. Continuous recording of central hemodynamic parameters was performed (Acknowledge/MP150, BIOPAC systems, Goleta, CA, USA). Arterial blood gases were sampled at baseline and at 15, 30, 45, 60, 75, and 90 min after bleeding start; they were analyzed in a GEM4000 (Instrumental Laboratory, Lexington, MA, USA). The experiment continued for 90 min (counted as survival) or was stopped if SBP (systemic blood pressure) dropped below 40 mmHg or if MAP (mean arterial pressure) dropped below 20 mmHg (defined as non-survival).

### Statistical analysis

An a priori power calculation was not performed due to the absence of pilot studies or published effect data. Survival was illustrated using a Kaplan–Meier curve. A Logrank test was performed to compare the survival of the groups. Normal distribution of continuous data was analyzed using the Shapiro–Wilk test. Accumulated bleeding volume was analyzed using mixed ANOVA with the factor group and time (repeated) and their interaction, followed by Tukey’s multiple comparison test between the groups if the interaction was statistically significant. Hemodynamics, including RITA blood flow and arterial blood variables, were analyzed using a one-way ANOVA including the factor group followed by Tukey’s multiple comparison test between the groups (if the group factor was statistically significant in the ANOVA) at time points of 10 min (for hemodynamics) and 15 min (for arterial blood variables) after start of bleeding. Beyond these time points, there was an increasing amount of missing data due to mortality.

Bleeding volume, RITA blood flow, hemodynamics, and arterial blood data are presented as means (95% confidence interval), and *p* < 0.05 was considered statistically significant. Statistical analysis was performed using Graph Pad Prism (version 9.5.1, GraphPad Software, LCC, San Diego, USA).

## Results

A total of 37 pigs were used in this study. Of these, 25 were included in the final analysis, and 12 were used for either method development (*N* = 8) or were excluded due to methodological failures (*N* = 4). In the latter group, the vena cava sheath became occluded in one animal, and in three animals, sheaths slipped out of the vena cava, femoral vein, or splenic vein. The included animals were randomly allocated to REBOA (*N* = 6), REBOA + REBOVC (*N* = 6), 4BO (*N* = 6), and control groups (*N* = 7).

### Survival and accumulated bleeding volume

**Fig. 2:**
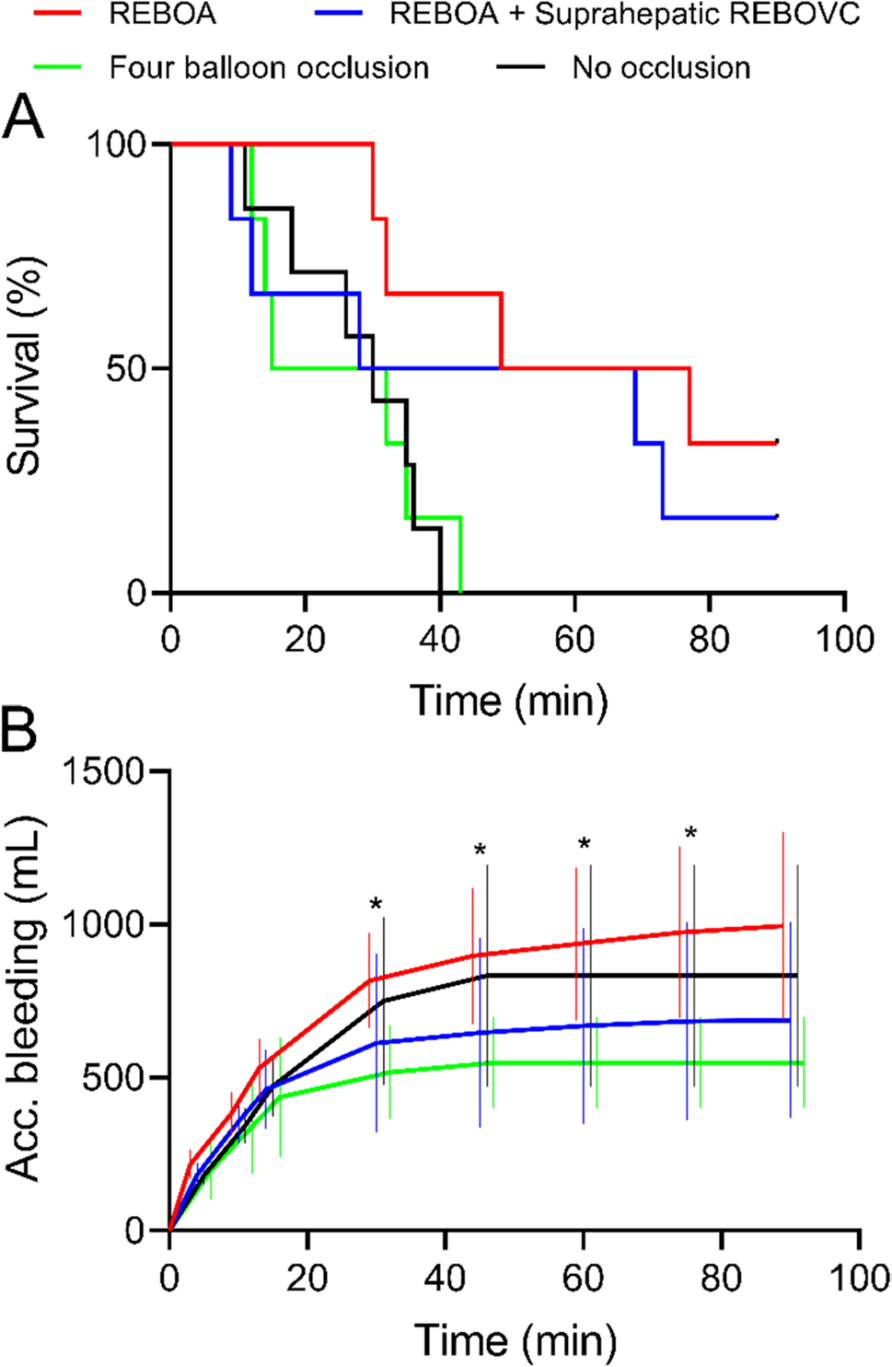
Survival (**A**) and accumulated bleeding volume (**B**) in anesthetized pigs randomized to either supraceliac resuscitative endovascular balloon occlusion of the aorta (REBOA, *N* = 6); REBOA + suprahepatic resuscitative endovascular balloon occlusion of the inferior vena cava (REBOVC, *N* = 6); REBOA + supra and infrahepatic REBOVC + endovascular portal vein balloon occlusion (four balloon occlusion,* N* = 6); or no occlusion (*N* = 7), started between time − 5 min and 0 min and subjected to free retrohepatic inferior vena cava bleeding (after time 0 min). Data are expressed as means with a 95% confidence interval. * indicates *P* < 0.05 between the REBOA group and the four balloon occlusion group at the respective time points

There was a difference in survival between the REBOA group (median survival time 63 min) and the 4BO group (median survival time 24 min, *P* = 0.02) and between the REBOA group and the control group (median survival time 30 min, *P* = 0.02; Fig. 2A). The median survival time in the REBOA + REBOVC group was 49 min (*P* > 0.05; Fig. 2A).

There was a difference in accumulated bleeding volume between the groups over time (*P* < 0.01 for the interaction between time and group, Fig. 2B). After 5, 10, and 15 min of bleeding, the accumulated bleeding volumes were comparable in all groups (*P* > 0.05). After 30 min and up to 75 min of bleeding, the bleeding volume was higher in the REBOA group compared to the 4BO group (*P* < 0.05); however, no other groups differed at any specific time points (*P* > 0.05, Fig. 2B).

### Hemodynamics

**Fig. 3:**
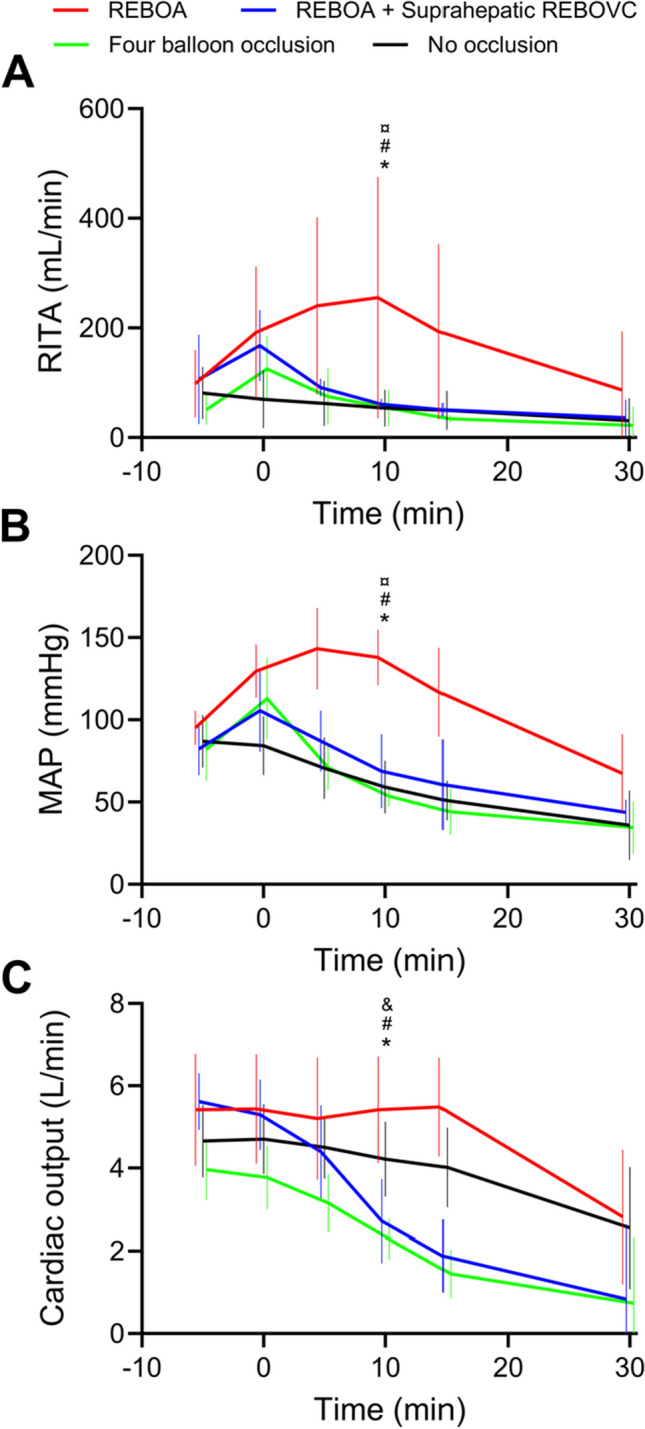
Blood flow in right internal thoracic artery (RITA, **A**), mean arterial systemic pressure (MAP, **B**), and cardiac output (**C**) in anesthetized pigs randomized to either supraceliac resuscitative endovascular balloon occlusion of the aorta (REBOA, *N* = 6); REBOA + suprahepatic resuscitative endovascular balloon occlusion of the inferior vena cava (REBOVC, *N* = 6); REBOA + supra and infrahepatic REBOVC + endovascular portal vein balloon occlusion (four balloon occlusion, *N* = 6); or no occlusion (*N* = 7) started between time − 5 min and 0 min and subjected to free retrohepatic inferior vena cava bleeding (after time 0 min). Data are expressed as means with a 95% confidence interval. *, #, and ¤ indicate *P* < 0.05 between the REBOA group and four balloon occlusion, REBOA + suprahepatic REBOVC group, and no occlusion group, respectively, at 10 min after start of bleeding. & indicates *P* < 0.05 between the four balloon occlusion group and no occlusion group, at 10 min after start of bleeding

Inflation of REBOA, REBOA + REBOVC, and 4BO provoked initially increased RITA blood flow and MAP; however, RITA blood flow and MAP were higher in the REBOA group compared with the other groups after the first 10 min of bleeding (*P* < 0.05, Fig. 3A, B). Upon inflation of the occlusion balloons and start of bleeding, CO started to decrease in the REBOA + REBOVC group and the 4BO group and was lower in these groups compared with the REBOA group after 10 min of bleeding (*P* < 0.05, Fig. 3C). CO in the no occlusion group did not differ significantly from the REBOA group (Fig. 3C). Over time, the RITA blood flow, MAP, and CO successively decreased in all groups, but a low RITA blood flow was present in all animals until death or at the end of the observation period (data not shown).

### Arterial blood variables

After 15 min of bleeding, arterial pCO_2_ was higher and arterial pH was lower in the no occlusion group compared to the other groups (*P* < 0.05, Table 1). Arterial lactate concentrations were higher in the REBOA (*P* < 0.05) and 4BO (*P* < 0.05) groups after 15 min of bleeding compared with the no occlusion group (Table 1). Over time, arterial lactate concentrations increased in all groups (Table 1 and data not shown). Arterial pO2 and hemoglobin concentration were similar in all groups (Table 1).

**Table 1:** Arterial blood gas variables in anesthetized pigs randomized to either supraceliac resuscitative endovascular balloon occlusion of the aorta (REBOA, *N* = 6); REBOA + suprahepatic resuscitative endovascular balloon occlusion of the inferior vena cava (REBOVC, *N* = 6); REBOA + supra and infrahepatic REBOVC + endovascular portal vein balloon occlusion (four balloon occlusion, *N* = 6); or no occlusion (*N* = 7) started between time − 5 min and 0 min and subjected to free retrohepatic inferior vena cava bleeding (after time 0 min)

Variable	− 5 min	15 min	30 min
Arterial pO_2_ (kPa)			
REBOA	11.2 (9.6–12.8) (*N* = 6)	13.5 (10.3–16.6) (*N* = 6)	14.4 (11.8–17.0) (*N* = 6)
REBOA + REBOVC	11.4 (9.1–13.7) (*N* = 6)	13.0 (10.0–16.0) (*N* = 4)	12.2 (8.4–16.0) (*N* = 3)
No occlusion	11.3 (10.4–13.4) (*N* = 7)	11.1 (9.4–12.8) (*N* = 6)	11.8 (9.2–14.3) (*N* = 4)
Four balloon occlusion	12.3 (11.0–13.6) (*N* = 6)	13.6 (9.3–18.0) (*N* = 4)	14.6 (8.6–20.6) (*N* = 3)
Arterial pH			
REBOA	7.43 (7.45–7.6) (*N* = 6)	7.65 (7.57–7.72)^¤^ (*N* = 6)	7.65 (7.58–7.72) (*N* = 6)
REBOA + REBOVC	7.50 (7.46–7.54) (*N* = 6)	7.71 (7.61–7.81)" (*N* = 4)	7.73 (7.68–7.78) (*N* = 3)
No occlusion	7.50 (7.45–7.55) (*N* = 7)	7.52 (7.48–7.57)^¤^ " ^&^ (*N* = 6)	7.49 (7.45–7.54) (*N* = 4)
Four balloon occlusion	7.56 (7.46–7.66) (*N* = 6)	7.75 (7.73–7.77)^&^ (*N* = 4)	7.72 (7.67–7.77) (*N* = 3)
Arterial pCO_2_ (kPa)			
REBOA	5.2 (4.8–5.5) (*N* = 6)	2.9 (2.5–3.4)^¤^ (*N* = 6)	2.5 (2.2–2.8) (*N* = 6)
REBOA + REBOVC	5.0 (4.7–5.3) (*N* = 6)	2.4 (2.0–2.7)" (*N* = 3)	2.2.(1.7–2.8) (*N* = 3)
No occlusion	5.2 (4.8–5.6) (*N* = 7)	4.7 (4.4–5.1)^¤^ "^&^ (*N* = 6)	4.4 (3.3–5.3) (*N* = 4)
Four balloon occlusion	4.8 (3.8–5.8) (*N* = 6)	2.5 (2.2–2.8)^&^ (*N* = 4)	2.5 (2.0–3.0) (*N* = 3)
Arterial lactate (mmol/L)			
REBOA	1.8 (1.3–2.3) (*N* = 6)	4.7 (3.3–6.0)^¤^ (*N* = 6)	6.6 (4.9–8.4) (*N* = 6)
REBOA + REBOVC	1.4 (1.2–1.7) (*N* = 6)	4.5 (1.4–7.5) (*N* = 4)	6.3 (2.5–10.0) (*N* = 3)
No occlusion	1.9 (1.0–2.7) (*N* = 7)	2.3 (1.4–3.2)^¤ &^ (*N* = 6)	5.3 (3.0–7.5) (*N* = 4)
Four balloon occlusion	2.8 (1.8–3.7) (*N* = 6)	5.3 (3.5–7.0)^&^ (*N* = 4)	7.0 (3.0–11.1) (*N* = 3)
Arterial Hb (g/L)			
REBOA	77 (71–84) (*N* = 6)	85 (75–94) (*N* = 6)	76 (68–84) (*N* = 6)
REBOA + REBOVC	74 (68–79) (*N* = 6)	71 (52–90) (*N* = 3)	67 (51–82) (*N* = 3)
No occlusion	75 (68–82) (*N* = 7)	74 (66–81) (*N* = 6)	73 (60–86) (*N* = 4)
Four balloon occlusion	81 (72–91) (*N* = 6)	78 (53–104) (*N* = 4)	74 (38–111) (*N* = 3)

Data are expressed as means (95% confidence interval)

¤ Statistically significant difference between REBOA group and no occlusion group

" Statistically significant difference between REBOA + REBOVC group and no occlusion group

^&^ Statistically significant difference between no occlusion group and four balloon occlusion group

## Discussion

In this study, we describe an endovascular Heaney maneuver (4BO), i.e., endovascular total hepatic isolation, and compared this with other potential methods for temporary circulatory stabilization and bleeding control of the retrohepatic space. Contrary to our hypothesis, REBOA was found to be associated with the highest survival and the best hemodynamic stability despite a large bleeding volume, whereas 4BO was comparable to the no occlusion group regarding these variables.

There appears to be an interplay between bleeding volume, the volume of venous blood congestion in the lower body, and the central blood volume that results in a certain effective circulating volume, which we believe is an important determinant for survival time. Our data indicate that hemodynamic stabilization (by REBOA) via centralization of blood volume has more impact on short-term survival in this study than the accumulated bleeding volume, although bleeding control in a clinical scenario is still of utmost importance. Supraceliac REBOA mechanically obstructs aortic blood flow thus hindering the inflow of blood to the lower body and, in addition, causes the release of vasoactive substances to the area proximal to the occluded aorta [18, 19].

The application of supra and/or infrahepatic REBOVCs and/or portal vein occlusion obstructs venous return at various levels. Previous data have suggested that a concomitant REBOA placed prior to placement of the venous occlusion balloons limits the effects of the venous balloons on lower body venous congestion and the achievement of acceptable hemodynamic stability [20]. In the present study, the addition of REBOVC to REBOA resulted in lower mean MAP and CO levels, possibly secondary to reduced venous return. Interestingly, these hemodynamic differences did not result in a statistically significant difference in survival between the REBOA group and the REBOA + REBOVC group. However, in the 4BO group, the addition of infrahepatic and portal vein occlusions to REBOA + REBOVC resulted in a survival comparable to the control group. We speculate that the low survival and relatively worse hemodynamic stability in the 4BO group may be associated with the collateral inflow of blood to the lower body (shown in this study as increased RITA blood flow by REBOA) and possibly reduced venous return by the three venous occlusion balloons in the 4BO group.

The complete arterial collateral flow during supraceliac REBOA is still not completely understood, and previous data are contradictory [21–23]. Grupp et al. considered the internal thoracic arteries to be the major collateral suppliers during supraceliac REBOA [24]. Accordingly, in their retrospective review of CT scans in human trauma patients, Wasicek et al. showed collateral blood flow from the internal thoracic-, the superior epigastric- and the intercostal arteries to the inferior epigastric- and external iliac arteries, bypassing supraceliac REBOA [21]. In contrast, in a porcine hemorrhagic shock model by Hoehn et al., an absence of collateral antegrade and retrograde blood flow during supraceliac REBOA was found [22]. The data in the present study indicate that there is a continuous arterial blood flow from the thoracic cavity via the internal thoracic arteries to the abdominal cavity, bypassing supraceliac REBOA. The data also suggest that this collateral blood flow is directly related to perfusion pressure and CO, but that the arterial collateral blood flow is present until circulatory collapse. This collateral blood flow will contribute to lower body congestion when applying venous occlusions. It must be stressed that a supraceliac REBOA may not control hepatic blood inflow via the hepatic artery as efficiently as a vessel clamp on this vessel. There is some evidence for a collateral flow bypassing the supraceliac REBOA to the hepatic artery and thus contributing to the persistent bleeding seen in the first 15 min in all groups. A traditional Pringle maneuver might more completely stop the hepatic blood flow via the hepatic artery and thereby more efficiently reduce retrohepatic bleeding [21].

Paradoxically in the present study, total accumulated bleeding volume was inversely correlated to survival. Centralizing the blood volume is probably more important than blood loss via bleeding for an acceptable effective circulating volume to increase survival in the short term; however, definitive bleeding control must be prioritized in a clinical case. Larger accumulated bleeding is thus tolerated in the REBOA group, whereas less bleeding is tolerated in the 4BO group. Previous data on the effect of REBOA on venous truncal bleeding are heterogenous but point towards a benefit from REBOA [25–27].

No significant differences in bleeding volume between the four groups were seen during the first 15 min of our study. Contrary to our hypothesis, the present study cannot therefore conclude that the REBOA + REBOVC and 4BO methods actually decreased bleeding from the retrohepatic inferior vena cava. This may be an important finding and may be explained by the complex vessel anatomy of the retrohepatic space. The inferior phrenic veins empty into the inferior vena cava at the level of the hepatic veins, and about eight small, but still significant, venous vessels also drain into the retrohepatic region [28]. It may be that these vessels, in combination with the arterial collateral blood flow, are the reason why the REBOA + REBOVC, and in particular the 4BO did not efficiently lower the bleeding volume. In contained retrohepatic bleeding, there may be a tamponing effect of the hematoma in the retrohepatic space which may reduce and hinder continued extensive bleeding. This potential effect was not present in the model in our study.

It must be acknowledged that the results in our study may not be entirely translatable to humans but still adds important knowledge, e.g., on the impact of differences in collateral anatomy between pigs and humans on physiology in REBOA ± REBOVC research. Due to anatomical differences, humans may tolerate REBOVC, and in particular 4BO, hemodynamically better than pigs. The Heaney maneuver has been used in humans for decades, and the combined REBOA + REBOVC has been reported in a few case reports, although mostly in the treatment of infrahepatic vena cava injuries [8, 9, 13]. Thus, there seems to be a discrepancy between our results and the clinical human application which may partly be explained by the differences in collateral supply between experimental animals and humans, e.g., the presence of an azygos vein [4, 5, 29–31]. The azygos vein provides an important venous collateral pathway between the IVC and the superior vena cava (SVC) in humans [32, 33].

**Fig. 4:**
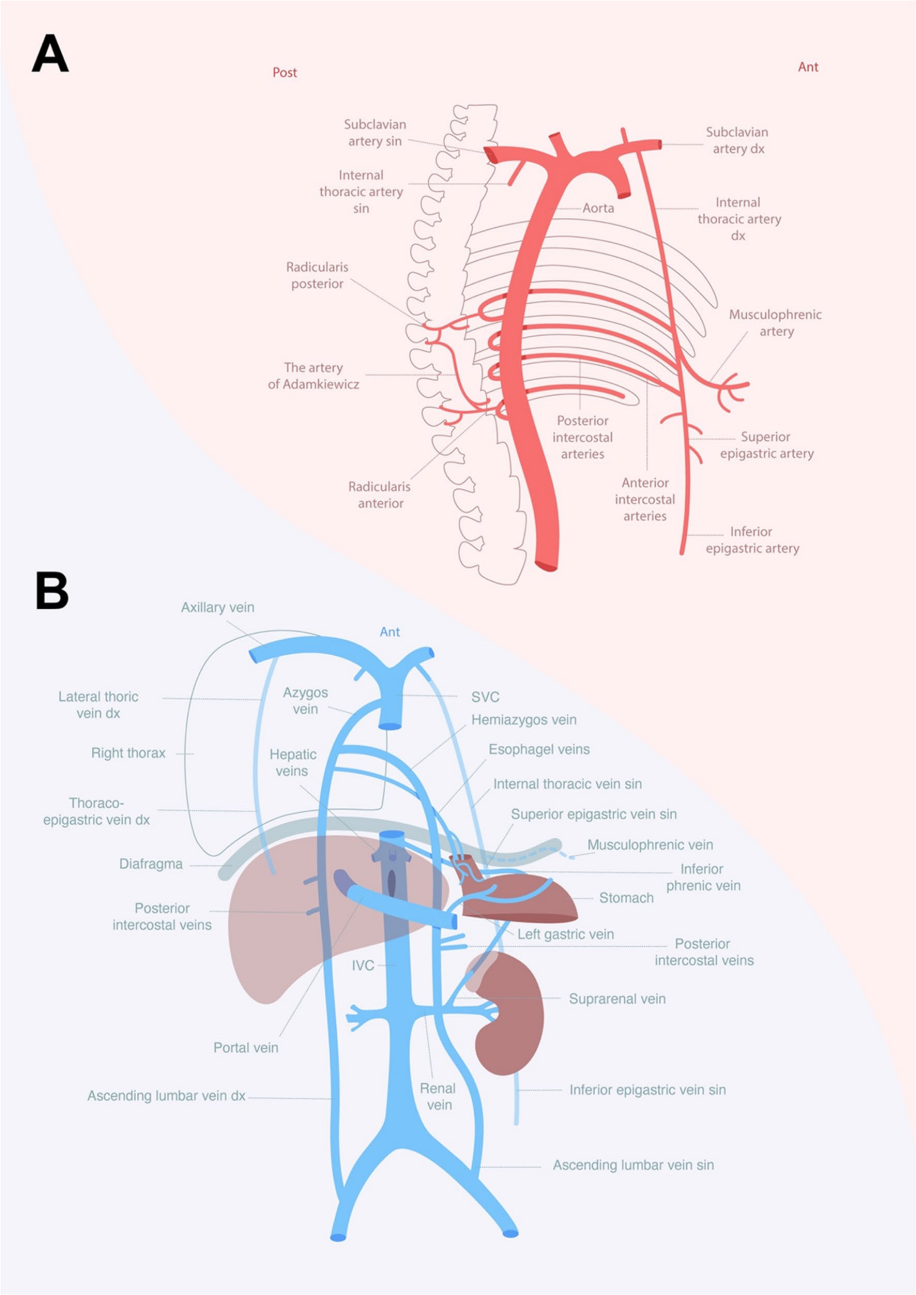
Illustration of the human arterial (**A**) and venous (**B**) collateral pathways between thorax and abdomen

There are other venous collaterals between the lower and upper body, including the hemiazygos vein, the paired inferior epigastric-, superior epigastric-, and internal thoracic veins emptying into the brachiocephalic veins, and the lateral thoracic veins emptying into the axillary veins [24, 32–34]. In addition to the azygos vein, these collaterals may counteract some of the venous lower body congestion when applying REBOVC in humans (Fig. 4).

In humans, the azygos vein, via the left gastric and the esophageal veins, is an alternative pathway to shunt splanchnic blood to the systemic circulation when the portal vein or hepatic circulation are blocked (Fig. 4) [32–35]. Pigs lack an azygos vein and therefore an efficient portosystemic collateral circulation, providing an anatomical basis for an extensive splanchnic venous congestion in portal occlusion [36, 37]. The poor tolerance of portal vein ligation in laboratory animals compared with humans has indeed been explained by the more extensive portosystemic venous collateral system in humans providing an escape route for splanchnic blood; this may contribute to better tolerance of portal vein occlusion and REBOVC in humans compared to pigs [29–31].

It may also be speculated that the difference (although not statistically significant) in survival between the REBOA + REBOVC group and the 4BO group in this study was additional deterioration of the inefficient portosystemic collateral circulation by the portal vein occlusion and/or the infrahepatic REBOVC. It may be important to avoid occlusion of potential venous collateral circulations in non-bleeding areas when using occlusion balloons, and even in open clamping, to maintain some venous return. In contrary, it may be important to occlude collateral venous circulation entering the area of injury to reduce bleeding. These considerations require a comprehensive understanding of the anatomy and function of venous collateral circulations and the effects of occluding them.

### Limitations

The study has some limitations. The pig model has some important anatomical differences compared with humans, as discussed above, which must be considered when interpreting the results and translating them to the human setting. Mean weight of the pigs was 29.1 kg, comparable to human children. The research group has for a long time collaborated with a farmer providing this special breed. The low mean weight of the pigs is not considered to have had any major impact of the results in this special study. The porcine model also employs free bleeding from the inferior cava vein and does not create a contained retrohepatic hematoma, thus not including a tamponing effect of the hematoma, which may reduce bleeding in humans. The advantage of our method was that the bleeding volume could be carefully measured. No additional resuscitative fluids, blood products, or vasopressors were given since we wanted to study the pure bleeding and hemodynamic effects of the interventions.

## Conclusions

An endovascular Heaney maneuver did not reduce accumulated retrohepatic vena cava bleeding nor produce hemodynamic stability and therefore did not contribute to improved survival compared with no occlusion. In contrast, supraceliac REBOA improved central hemodynamics and survival time, despite a large bleeding volume. The combination of supraceliac REBOA and suprahepatic REBOVC performed better than the endovascular Heaney maneuver but worse than supraceliac REBOA. These findings may be attributed to arterial collateral blood flow to the lower body, lower body venous congestion, and the lack of or interference with efficient portosystemic collaterals. Anatomical differences between pigs and humans must be considered when interpreting these results.

## Supplementary information

Below is the link to the electronic supplementary material.

Supplementary file1 (DOCX 31 KB)

## Data Availability

All datasets are stored by the corresponding author and available from the corresponding author on reasonable request.

## References

[1] Asensio JA, et al. Operative management and outcome of 302 abdominal vascular injuries. Am J Surg. 2000;180(6):528–33. 10.1016/s0002-9610(00)00519-5. 11182412 10.1016/s0002-9610(00)00519-5

[2] Khan MZ, Khan A, Mbebe DT, Bruce JL, Bekker W, Clarke DL. Despite major therapeutic advances, vena caval trauma remains associated with significant morbidity and mortality. World J Surg. 2022;46(3):577–81. 10.1007/s00268-021-06403-x. 35001138 10.1007/s00268-021-06403-x

[3] Stonko DP, et al. Contemporary management and outcomes of injuries to the inferior vena cava: a prospective multicenter trial from prospective observational vascular injury treatment. Am Surg. 2021; 31348211038556. 10.1177/00031348211038556. 10.1177/00031348211038556 34384266

[4] Klein SR, Baumgartner FJ, Bongard FS. Contemporary management strategy for major inferior vena caval injuries. J Trauma. 1994;37(1):35–41. 10.1097/00005373-199407000-00008. 8028056 10.1097/00005373-199407000-00008

[5] Kosola J, Brinck T, Leppäniemi A, Handolin L. Blunt abdominal trauma in a european trauma setting: need for complex or non-complex skills in emergency laparotomy. Scand J Surg. 2020;109(2):89–95. 10.1177/1457496919828244. 30782110 10.1177/1457496919828244

[6] Choi D, Kang BH, Jung K, Lim SH and Moon J. Risk factors and management of blunt inferior vena cava injury: a retrospective study. World J Surg. 2023. 10.1007/s00268-023-07110-5. 10.1007/s00268-023-07110-5 37423908

[7] Bui TD, Mills JL. Control of inferior vena cava injury using percutaneous balloon catheter occlusion. Vasc Endovascular Surg. 2009;43(5):490–3. 10.1177/1538574409339939. 19628517 10.1177/1538574409339939

[8] Ordonez CA, Herrera-Escobar JP, Parra MW, Rodriguez-Ossa PA, Puyana JC, Brenner M. A severe traumatic juxtahepatic blunt venous injury. J Trauma Acute Care Surg. 2016;80(4):674–6. 10.1097/ta.0000000000000979. 26808042 10.1097/TA.0000000000000979

[9] Bisulli M, Gamberini E, Coccolini F, Scognamiglio G, Agnoletti V. Resuscitative endovascular balloon occlusion of vena cava: an option in managing traumatic vena cava injuries. J Trauma Acute Care Surg. 2018;84(1):211–3. 10.1097/ta.0000000000001707. 28930943 10.1097/TA.0000000000001707

[10] Rosenthal MD, Raza A, Markle S, Croft CA, Mohr AM, Smith RS. The novel use of resuscitative endovascular balloon occlusion of the aorta to explore a retroperitoneal hematoma in a hemodynamically unstable patient. Am Surg. 2017;83(4):337–40. 28424126

[11] Angeles AP, Agarwal N, Lynd C Jr. Repair of a juxtahepatic inferior vena cava injury using a simple endovascular technique. J Trauma. 2004;56(4):918–21. 15187764 10.1097/01.ta.0000084516.50653.c7

[12] Aseni P, Henry S, Grande AM, Fiore A, and Scalea TM. Lifesaving and emergency surgical procedures in trauma patients. In: Aseni P, Grande AM, Leppäniemi A, Chiara O, editors. The high-risk surgical patient. 1st ed. Cham: Springer; 2023. ch. 80, pp. 901–945.

[13] Howell EC, Kulkarni SS, Walker PF, Morrison JJ, Kundi R, Scalea TM. Endovascular balloon occlusion of the inferior vena cava in trauma: a single-center case series. J Am Coll Surg. 2023;236(2):e1–7. 10.1097/xcs.0000000000000436. 36165502 10.1097/XCS.0000000000000436

[14] Hörer T. Resuscitative endovascular balloon occlusion of the aorta (REBOA) and endovascular resuscitation and trauma management (EVTM): a paradigm shift regarding hemodynamic instability. Eur J Trauma Emerg Surg. 2018;44(4):487–9. 10.1007/s00068-018-0983-y. 30084088 10.1007/s00068-018-0983-y

[15] Kilkenny C, Browne WJ, Cuthill IC, Emerson M, Altman DG. Improving bioscience research reporting: the ARRIVE guidelines for reporting animal research. PLoS Biol. 2010;8(6):e1000412. 10.1371/journal.pbio.1000412. 20613859 10.1371/journal.pbio.1000412 PMC2893951

[16] Wikström MB, Smårs M, Karlsson C, Stene Hurtsén A, Hörer TM, Nilsson KF. A randomized porcine study of the hemodynamic and metabolic effects of combined endovascular occlusion of the vena cava and the aorta in normovolemia and in hemorrhagic shock. J Trauma Acute Care Surg. 2021;90(5):817–26. 10.1097/ta.0000000000003098. 33496552 10.1097/TA.0000000000003098 PMC8081444

[17] Wikström MB, Åström J, Stene Hurtsén A, Hörer TM, Nilsson KF. A porcine study of ultrasound-guided versus fluoroscopy-guided placement of endovascular balloons in the inferior vena cava (REBOVC) and the aorta (REBOA). Trauma Surg Acute Care Open. 2023;8(1):e001075. 10.1136/tsaco-2022-001075. 37205275 10.1136/tsaco-2022-001075 PMC10186488

[18] Stokland O, Miller MM, Ilebekk A, Kiil F. Mechanism of hemodynamic responses to occlusion of the descending thoracic aorta. Am J Physiol. 1980;238(4):H423–9. 10.1152/ajpheart.1980.238.4.H423. 7377312 10.1152/ajpheart.1980.238.4.H423

[19] Gelman S, Bredle DL, Bradley WE, Cain SM. Angiotensin and alpha-adrenoceptor activation play a role in hemodynamic response to aortic cross-clamping. Am J Physiol. 1990;259(1 Pt 2):H68-73. 10.1152/ajpheart.1990.259.1.H68. 2165366 10.1152/ajpheart.1990.259.1.H68

[20] Wikström MB, Krantz J, Hörer TM, Nilsson KF. Resuscitative endovascular balloon occlusion of the inferior vena cava is made hemodynamically possible by concomitant endovascular balloon occlusion of the aorta-a porcine study. J Trauma Acute Care Surg. 2020;88(1):160–8. 10.1097/ta.0000000000002467. 31397743 10.1097/TA.0000000000002467

[21] Wasicek PJ, et al. Assessment of blood flow patterns distal to aortic occlusion using CT in patients with resuscitative endovascular balloon occlusion of the aorta. J Am Coll Surg. 2018;226(3):294–308. 10.1016/j.jamcollsurg.2017.12.005. 29248608 10.1016/j.jamcollsurg.2017.12.005

[22] Hoehn MR, et al. Aortic branch vessel flow during resuscitative endovascular balloon occlusion of the aorta. J Trauma Acute Care Surg. 2019;86(1):79–85. 10.1097/ta.0000000000002075. 30252777 10.1097/TA.0000000000002075

[23] Izawa Y, Hishikawa S, Matsumura Y, Nakamura H, Sugimoto H, Mato T. Blood flow of the venous system during resuscitative endovascular balloon occlusion of the aorta: noninvasive evaluation using phase contrast magnetic resonance imaging. J Trauma Acute Care Surg. 2020;88(2):305–9. 10.1097/ta.0000000000002557. 31804421 10.1097/TA.0000000000002557

[24] Grupp G, Grupp IL, Spitz HB. Collateral vascular pathways during experimental obstruction of aorta and inferior vena cava. Am J Roentgenol Radium Ther Nucl Med. 1965;94:159–71. 14281851

[25] Glaser JJ, Neidert LE, Morgan CG, Brenner M, Stigall KS, Cardin S. Resuscitative endovascular balloon occlusion of the aorta for thoracic trauma in the setting of platelet dysfunction: a translational swine study. J Trauma Acute Care Surg. 2020;89(4):708–15. 10.1097/ta.0000000000002882. 32649613 10.1097/TA.0000000000002882

[26] Russo RM, et al. Extending the golden hour: partial resuscitative endovascular balloon occlusion of the aorta in a highly lethal swine liver injury model. J Trauma Acute Care Surg. 2016;80(3):372–8. 10.1097/ta.0000000000000940. 26670114 10.1097/TA.0000000000000940

[27] Lallemand MS, et al. Resuscitative endovascular balloon occlusion of the aorta for major abdominal venous injury in a porcine hemorrhagic shock model. J Trauma Acute Care Surg. 2017;83(2):230–6. 10.1097/ta.0000000000001548. 28459798 10.1097/TA.0000000000001548

[28] Fullen WD, McDonough JJ, Popp MJ, Altemeier WA. Sternal splitting approach for major hepatic or retrohepatic vena cava injury. J Trauma. 1974;14(11):903–11. 4422259 10.1097/00005373-197411000-00001

[29] Delva E, et al. Hemodynamic and biochemical monitoring during major liver resection with use of hepatic vascular exclusion. Surgery. 1984;95(3):309–18. 6701787

[30] Delva E, Camus Y, Paugam C, Parc R, Huguet C, Lienhart A. Hemodynamic effects of portal triad clamping in humans. Anesth Analg. 1987;66(9):864–8. 3619092

[31] Delva E, et al. Vascular occlusions for liver resections. Operative management and tolerance to hepatic ischemia: 142 cases. Ann Surg. 1989;209(2):211–8. 10.1097/00000658-198902000-00012. 2916865 10.1097/00000658-198902000-00012 PMC1493903

[32] Piciucchi S, et al. The azygos vein pathway: an overview from anatomical variations to pathological changes. Insights Imaging. 2014;5(5):619–28. 10.1007/s13244-014-0351-3. 25171956 10.1007/s13244-014-0351-3 PMC4195836

[33] Sharma M, Rameshbabu CS. Collateral pathways in portal hypertension. J Clin Exp Hepatol. 2012;2(4):338–52. 10.1016/j.jceh.2012.08.001. 25755456 10.1016/j.jceh.2012.08.001 PMC3940321

[34] Netter FH, Hansen JT, editors. Atlas of human anatomy. 6th ed. Philadelphia: Elsevier Saunders; 2014. p. 531.

[35] Douzdjian V, Broughan TA. Spontaneous splenic rupture during total vascular occlusion of the liver. Br J Surg. 1995;82(3):406–7. 10.1002/bjs.1800820343. 7796027 10.1002/bjs.1800820343

[36] Crick SJ, Sheppard MN, Ho SY, Gebstein L, Anderson RH. Anatomy of the pig heart: comparisons with normal human cardiac structure. J Anat. 1998;193(Pt 1):105–19. 10.1046/j.1469-7580.1998.19310105.x. 9758141 10.1046/j.1469-7580.1998.19310105.x PMC1467827

[37] Sack WO. Essentials of pig anatomy. Veterinary Textbooks; 1982, p. 192.

