# Supplementary material for: The effect of an endovascular Heaney maneuver to achieve total hepatic isolation on survival, hemodynamic stability, retrohepatic bleeding, and collateral flow in a porcine model
Source: Eur J Trauma Emerg Surg. 2024 Mar 8;50(4):1547–57. doi: 10.1007/s00068-024-02482-2 (PMC11458705; doi:10.1007/s00068-024-02482-2)
Supplement: Supplementary file 1 — Supplementary file1 (DOCX 31 KB) [file 68_2024_2482_MOESM1_ESM.docx]

**The ARRIVE guidelines 2.0 Author checklist**

**These items are the basic minimum to include in a manuscript. Without this information, readers and reviewers cannot assess the reliability of the findings.**

**Study design**

**1For each experiment, provide brief details of study design including:**

**a. The groups being compared, including control groups. If no control group has been used, the rationale should be stated.**

**b. The experimental unit (e.g. a single animal, litter, or cage of animals).**

**1a)** Four groups compared:

1. REBOA (N=6)
2. REBOA + suprahepatic REBOVC (N=6)
3. REBOA + supra- and infrahepatic REBOVC + portal vein occlusion (N=6)
4. No occlusion = control group (N=7)

**1b)** Single animal (N=6-7/group)

**Sample size**

**2a. Specify the exact number of experimental units allocated to each group, and the total number in each experiment. Also indicate the total number of animals used.**

**b. Explain how the sample size was decided. Provide details of any a priori sample size calculation, if done.**

**2a)** Total animals used = 37. Included: 25 (6+ 6+6+7). Excluded: 12

**2b)** An *a priori* power calculation was not executed due to the abscence of pilotstudies or published effect data.

**Inclusion and exclusion criteria**

**3a. Describe any criteria used for including and excluding animals (or experimental units) during the experiment, and data points during the analysis. Specify if these criteria were established a priori. If no criteria were set, state this explicitly.**

**b. For each experimental group, report any animals, experimental units or data points not included in the analysis and explain why. If there were no exclusions, state so.**

**c. For each analysis, report the exact value of n in each experimental group.**

**3a)** Animals not considered to represent normal baseline hemodynamic parameters at start due to e.g failures during surgical preparation with bleeding was not included. Nor were animals which had unintended not experimental-related incidents such as catheters slipping out of large vessels included, since the bleeding affected the hemodynamic parameters and they were therefore not considered representative to include in the study. During the study, it was predetermined to stop the experiments if systemic blood pressure dropped < 40 mmHg or mean arterial pressure < 20 mmHg.

**3b)** 8 animals were used for method development, 3 animals had catheters that slipped out of the IVC, the femoral vein or the splenic vein which caused bleeding and affected the animals hemodynamically. 1 animal had the vena cava catheter occluded, flushing of the catheter was not made according to the protocol and bleeding stopped due to methodological failures. The animal was therefore not considered representative to be included.

**3c)**

REBOA (N=6)

REBOA + suprahepatic REBOVC (N=6)

REBOA + supra- and infrahepatic REBOVC + portal vein occlusion (N=6)

No occlusion = control group (N=7)

**Randomisation**

**4a. State whether randomisation was used to allocate experimental units to control and treatment groups. If done, provide the method used to generate the randomisation sequence.**

**b. Describe the strategy used to minimise potential confounders such as the order of treatments and measurements, or animal/cage location. If confounders were not controlled, state this explicitly.**

**4a)** Randomization was made by blindly drawing lots from a ballot to one of the four groups : 1) REBOA, 2) REBOA + REBOVC 3) REBOA + infra- and suprahepatic REBOVC or 4) No occlusion = control group.

**4b)** To minimise confounding the randomisation was performed blindly and by different persons in the researchteam. One person prepared the ballots and another person was blindly drawing the ballots.

**Blinding5**

**Describe who was aware of the group allocation at the different stages of the experiment (during the allocation, the conduct of the experiment, the outcome assessment, and the data analysis).**

**5)** Before the ballots were drawn and allocation performed, it was unknown to the whole researchteam which intervention would be performed in each animal. After the randomisation was performed, the whole researchteam was aware of the allocation/ which intervention that certain animal was going to have.

Since everyone in the researchteam at lab had to be involved in the performance of the experiments it was not possible to conduct these complicated experiments blindly. Outcome assessment and data analysis was performed mainly by one person and blinded to the others in the researchteam, until the results were presented.

**Outcome measures**

**6a. Clearly define all outcome measures assessed (e.g. cell death, molecular markers, or behavioural changes).**

**b. For hypothesis-testing studies, specify the primary outcome measure, i.e. the outcome measure that was used to determine the sample size.**

**6a)** Survival and retrohepatic bleeding were the primary outcomes.

The impact on collateral flow in the right internal thoracic artery (RITA), hemodynamics (CO, MAP, SBP, HR, mCVP, EtCO2, distal aortaflow) and blood gas markers of metabolism (pH, pO2, pCO2, lactate, base excess, potassium, calcium) were secondary outcomes.

**6b)** An *a priori* power calculation of sample size was not executed due to the abscence of pilotstudies or published effect data.

**Statistical methods**

**7a. Provide details of the statistical methods used for each analysis, including software used.**

**b. Describe any methods used to assess whether the data met the assumptions of the statistical approach, and what was done if the assumptions were not met.**

**7a)** Comparison of survival between the groups was performed with Logrank test and illustrated by a Kaplan Mayer curve.

Shapiro Wilks test was used to check for normal distribution. Accumulated bleeding volume was analyzed using mixed ANOVA with the factor group and time (repeated) and their interaction, followed by Tukey´s multiple comparison test between the groups if the interaction was statistically significant. Hemodynamics including RITA blood flow and arterial blood variables were analyzed using a

One-way ANOVA fincluding the factor group followed by Tukey ´s multiple comparisons test between the groups (if the group factor was statistically significant in the ANOVA) at the time point 10 min (for hemodynamics) and 15 min (for arterial blood variables) after bleeding start. Beyond these time points there was an increasing amount of missing data due to mortality.

Bleeding volume, RITA blood flow, hemodynamics and arterial blood data are presented as means (95% confidence interval) and p< 0.05 was considered statistically significant.

Software used:

Graph Pad Prism, version 9.5.1 (GraphPad Software, LCC, San Diego, USA).

**7b)** See answer 7a.

**Experimental animals**

**8a. Provide species-appropriate details of the animals used, including species, strain and substrain, sex, age or developmental stage, and, if relevant, weight.**

**b. Provide further relevant information on the provenance of animals, health/immune status, genetic modification status, genotype, and any previous procedures.**

**8a)** The animals were cross-breed between England Yorkshire, Swedish conutry breed and Hampshire, aged 3 months, gender ratio 1:1 and mean weight 29.1 kg, range 23-33 kg.

**8b)** The animals were bred at a local farmer with which the university animal research laboratory has a long history of cooperation. The animals were supplied with food and drink by the farmer before transport to the laboratory. The experiments are performed in the animal research laboratory the same day as they are fetched from the farm. The animals chosen for research are all healthy with no previously known defects or diseases, nor area ny gene modifications performed.

**Experimental procedures**

**9For each experimental group, including controls, describe the procedures in enough detail to allow others to replicate them, including:**

**a. What was done, how it was done and what was used.**

**b. When and how often.**

**c. Where (including detail of any acclimatisation periods).**

**d. Why (provide rationale for procedures).**

**9 a-e) For all experimental animals:**

An intramuscular injection of 240 mg azaperone( 40 mg/ml, Virbac, Kolding, Denmark) was given before pickup to the laboratory. To induce anesthesia, a blend of zolazepam (6 mg/kg, Virbac) tiletamine (6 mg/kg, Virbac) and azaperone (4 mg/kg) was injected i.m at laboratory arrival. For maintainance of general anesthesia, continuous infusions of propofol (10 mg/kg/h, Fresenius Kabi) and remifentanil (0,5 µg/kg/min, Meda AB, Solna, Sweden) were administred. Atropine (1.5 mg i.m, Mylan, Stockholm, Sweden) was given prior to endotracheal intubation, followed by adjustment of the respiratory frequency to normoventilation by constant ventilation at tidal volume of 10 ml/kg. A solution of 5%glucose (1 ml/kg/h, Fresenius Kabi) were running throughout the experiment. Ringer`s acetate solution (10 ml/kg/h), Fresenius Kabi) was started at the initial preparations and stopped when the experimental retrohepatic bleeding started. Thermal blankets were used to keep body temperature at 37.5-39.5 °C To euthanize the animals after completion of the experiments, an i.v dose of potassium chloride 40 mmol was rapidly given.

Surgical preparation

In the right external jugular vein, a 10 Fr sheat was introduced and a 7.5 Fr Swan-Ganz arterial pulmonary catheter (Edward Lifesciences, Swan-Ganz CCOmbo, Irvine, CA) inserted. For sampling of arterial bloods, a 5 Fr sheath was placed in the right common carotid artery by open surgical exposure. To give drugs and fluids, a 7 Fr sheath was placed in the left external jugular vein. The right internal thoracic artery (RITA) was exposed by a parasternal incision at the level of 2^nd^ intercostal space, and a 3 mm probe (Transonic Systems Inc, NY, USA) for blood flow measurement was placed. By an incision in the left flank, the splenic vein was exposed, an 11 Fr sheath placed to prepare for the endovascular balloon occlusion of the portal vein. In the right groin, the right femoral artery was exposed, an 11 Fr sheath prepared for the REBOA catheter and the right femoral vein exposed and an 11 Fr sheath prepared for the suprahepatic REBOVC catheter. The left femoral vein was exposed and an 11 Fr sheath placed for the infrahepatic REBOVC catheter. In the right flank, an incision was made to expose and put an 11 Fr sheath into the retrohepatic part of vena cava. Through the same flank incision, a 10 mm probe (Transonic Systems Inc, NY, USA) was put on the distal aorta, between the renal arteries and the bifurcation, to measure the distal aortic blood flow. A urinary catheter was positioned in the urinary bladder by a small suprapubic incision.

Study protocol

When basic surgical preparations were finished, 5000 E Heparin i.v was given and 1 hour of rest followed. Randomization of the 25 animals to either of four groups: 1) supracoeliac REBOA (N=6) 2) Supracoeliac REBOA + suprahepatic REBOVC (N=6) 3) supracoeliac REBOA + infra- and suprahepatic REBOVC + portal vein occlusion (four-balloon-occlusion, 4BO) (N=6) and 4) no occlusion (control group) (N=7).

The aortic ballon was always inflated first, followed by the portal vein, the infrahepatic vena cava and lastly in the suprahepatic vena cava. Correct balloon position was verified by fluoroscopy. Due to our previous experience with the Equalizer™ balloon for vena cava occlusion, we opted to use the Equalizer™ (Boston Scientific, Ireland) for venous occlusion and for aortic occlusion either the ER-REBOA™ (Prytime Medical, USA) or the Tokai™ balloon (Tokai Medical Products, Japan). It took 5 minutes to inflate all balloons (-5 min from start of bleeding = time 0). Immediately after balloon inflation, the solution of Ringer`s acetate was stopped, 10 000 E Heparin was given, and bleeding started from the prepared 11 Fr catheter in retrohepatic vena cava placed at a level slightly below the heart. The pigs were placed in left lateral position and free bleeding was allowed into a plastic bag on a scale. Every minute the amount of blood in the plastic bag was recorded. The catheter for bleeding was flushed with 1-2 ml saline when the flow decreased, to keep the catheter open. The exact amount of flushed fluid was extracted from the total bleeding amount. The blood flow in RITA and the distal aorta were recorded with one-minute intervals. Continuous recording of hemodynamic parameters were performed and arterial blood gases sampled at baseline and 15-, 30-, 45-, 60-, 75-, and 90 minutes after bleeding start. The experiment continued for 90 minutes or was stopped if SBP (Systemic Blood Pressure) dropped below 40 mmHg or MAP (Mean Arterial Pressure) below 20 mmHg.

**Results**

**10For each experiment conducted, including independent replications, report:**

1. **Summary/descriptive statistics for each experimental group, with a measure of variability where applicable (e.g. mean and SD, or median and range).**

**10a)**

**Results**

In total, 37 pigs were used in this study, of which 25 were included in the final analysis and 12 animals were used for either method development (N=8) or were excluded due to methodological failures with hemodynamic effects and therefore considered not representative (N=4). One animal had the vena cava catheter occluded and three had introducers that slipped out of the vena cava, femoral vein or splenic vein.

Survival

A significant difference in median survival times were seen between the REBOA group (63 minutes, Log-rank (Mantel-Cox test), the 4BO group (24 minutes, p = 0.02) and the control group (30 minutes, p = 0.02). Although not statistically significant, there was a tendency to longer survival in the REBOA + REBOVC group (median 49 minutes) compared to the 4BO group and the control group (25 minutes and 19 minutes respectively) (p > 0.05).

Retrohepatic bleeding

After 5, 10 and 15 minutes of bleeding, the mean accumulated bleeding volumes were comparable in all groups, and no significant differences in bleeding volumes were found

(p > 0.05, unpaired t-tests).

At 30 minutes (p=0.01), 45 minutes(p=0.01), and 90 minutes (p=0.01) the bleeding volumes were significantly higher in the REBOA group, compared to the 4BO group (p=0.01).

Mean accumulated bleeding volumes and 95 % confidence interval (CI) at 15 minutes and 90 minutes, respectively:

1) REBOA 531 ml (431-631) and 995 ml (673-1316),

2) REBOA + REBOVC 460 ml (325-595) and 687 ml (351-1022),

3) Four-balloon-occlusion 436 ml (229-643) and 548 ml (391-705), and

4) No occlusion 472 ml (381-564) and 832 ml (496-1168).

RITA flow

The REBOA group had a significant higher mean RITA blood flow than all the other groups at 5 and 10 minutes. At 15 minutes RITA flow was still significantly higher in the REBOA group (193 ml/min [95 % CI 34-353 ml/min] compared to the 4BO group (34 ml/min [95 % CI 28-41 ml/min, p= 0.04] and the control group (50 ml/min [95% CI 14-85 ml/min, p= 0.03]. At 30 minutes, no significant differences were seen between the four groups. RITA flow remained in all animals until death.

Hemodynamics

At 15 min significantly higher CO was found in the REBOA group vs: REBOA + REBOVC group (p<0.001), the control group (p=0.03), and the 4BO group (p<0.001) and between the control group vs: the REBOA + REBOVC group (p<0.001), and the 4BO group (p=0.002). At 30 min no significant differences in CO were found between the groups.

At 5, 10, 15 and 30 min significantly higher Mean Arterial Pressure (MAP) was found in the REBOA group vs: the REBOA + REBOVC group (p<0.001, p< 0.001, p<0.001 and p=0.04 respectively), the control group (p< 0.001, p< 0.001, p< 0.001 and p=0.04) and the 4BO group (p< 0.001, p< 0.001, p< 0.001 and p= 0.05). MAP was still higher in the REBOA group vs the REBOA + REBOVC group at 45 min (p=0.008). After 45 min no significant differences in MAP were found.

Arterial Blood Gases

At any time, no significant differences in pO2 were found between the four groups. At 15 min and 30 min significantly higher pCO2 and lower pH were found in the control group vs: the REBOA group (No significant differences were found in pCO2 after 45 minutes. At 60 min art pH was significantly lower in the REBOA group vs the REBOA + REBOVC group (p=0.03) but no significant differences were found at 45, 75 or 90 minutes.

At 15 min significantly lower arterial lactate was seen in the control group vs: the REBOA group (p=0.023) and the 4BO group (p=0.011). After 30 min no significant differences in art.lactate were found. No significant differences in arterial Hb between the four groups were found after 15 min.

1. **If applicable, the effect size with a confidence interval.**

**10b)** See results in 10a) above.
